# Opioid-free anesthesia: comparable analgesia with potential superiority in safety and quality of recovery

**DOI:** 10.1186/s44158-025-00294-3

**Published:** 2025-10-16

**Authors:** Jan P. Mulier, Patrice Forget, Patricia Lavand’homme, Luc De Baerdemaeker, Hans D. de Boer

**Affiliations:** 1 Ghent University Hospital, Ghent, Belgium; 2 Faculty of Medicine and Health Sciences, Department of Basic and Applied Medical Sciences, University Ghent, Ghent, Belgium; 3 Aberdeen Centre for Arthritis and Musculoskeletal Health (Epidemiology Group), Institute of Applied Health Sciences, School of Medicine, Medical Sciences and Nutrition, Aberdeen, AB25 2ZD UK; 4 Anaesthesia department, NHS Grampian, Aberdeen, AB25 2ZD UK; 5 Université Catholique de Louvain, Department of Anesthesiology and Acute Postoperative & Transitional Pain Service, Brussels, Belgium; 6 Martini General Hospital Groningen, the Netherlands, Department of Anesthesiology and Pain Medicine, Groningen, Netherlands

**To the Editor,**

We read with great interest the systematic review and network meta-analysis by Tripodi et al. on opioid-free versus opioid-based anesthesia (OFA vs OBA) [1]. Their stated aim was to compare the effectiveness and safety of OFA regimens against opioid-based anesthesia (OBA).

However, the primary endpoint chosen to define “effectiveness” was restricted to postoperative pain, as if the purpose of anesthesia could be reduced to analgesia alone. Pain management cannot be limited to pain intensity alone; it must also include quality of recovery and the burden of adverse events. More importantly, the principal goal of anesthesia is to provide surgeons with optimal operating conditions while ensuring patient safety through stable organ perfusion and physiological support. This extends far beyond pain control, encompassing suppression of stress responses, modulation of the surgical inflammatory cascade, and preparation for safe recovery. Minimizing not only pain and postoperative nausea and vomiting (PONV), but also the wider range of complications that may follow surgery and anesthesia. Indeed, the benefits of OFA may extend throughout the entire perioperative period, influencing both physiological stability and patient-reported outcome measures (PROMs) [2].

Even in the absence of large randomized controlled trials (RCTs), there is broad consensus that multimodal anesthesia with reduced opioid use is preferable to opioid-based anesthesia alone, precisely to limit opioid-related adverse effects [3]. If reducing opioid exposure is already considered beneficial, why hesitate to proceed to a fully opioid-free intraoperative regimen, particularly when stable anesthesia is achieved just as effectively, if not more so? OFA is not simply the replacement of an opioid with a non-opioid analgesic; it aligns seamlessly with the concept of multimodal anesthesia.

The second pillar of the analysis was safety, but in practice this was assessed mainly through hemodynamic stability, where OFA proved superior. Other outcomes, such as PONV, were analyzed only as secondary endpoints. Here again OFA showed superiority, yet because these endpoints were not predefined as primary, they were excluded from the authors’ main conclusions.

It is also important to recognize the limitations of RCT methodology. RCTs are designed to address a single focused question under controlled conditions, typically exclude large patient groups, and often include relatively small cohorts. Their protocols may not reflect optimal daily practice. In contrast, retrospective analyses include all procedures, capture larger populations, and are better suited to evaluate safety and rare adverse events. This meta-analysis included 42 RCTs with 4666 patients, whereas a single retrospective bariatric cohort from our group already included 9246 patients at one center and demonstrated fewer adverse events with OFA [4].

Tripodi et al.’s findings confirm that while postoperative pain at 24 h did not differ significantly, OFA, especially combinations including ketamine, alpha-2 agonists, and lidocaine was associated with reduced PONV, decreased postoperative opioid requirements, and a lower incidence of intraoperative hypotension. If intraoperative opioids offer no benefit for postoperative pain control yet clearly increase the risk of side effects, their continued routine use in multimodal anesthesia is difficult to justify.

Furthermore, the discussion of OFA should not be confined to symptoms such as pain or nausea. During surgery, the systemic stress response is attenuated, but not abolished, by regional anesthesia [5, 6], and inflammatory activation persists. Consequently, large comparative studies often show little or no difference in major postoperative outcomes between regional and general anesthesia [7, 8]. This suggests that excessive inflammation remains an unmet need. OFA protocols including intravenous lidocaine and dexmedetomidine achieve the most substantial reductions in inflammatory markers, outperforming either drug alone as well as opioid-based regimens [9].

As Kehlet noted as early as 1985, “stress-free anesthesia does not exist, whatever dose of opioids you give” [10]. Indeed, opioids have never been shown to attenuate systemic inflammation. By contrast, lidocaine and alpha-2 agonists are potent anti-inflammatory agents, and their judicious intraoperative use may finally bring us closer to the long-sought goal of stress- and inflammation-free anesthesia.

Correct dosing, however, is critical. Several published studies overdosed (e.g. the POFA trial by Beloeil et al. [11], as we highlighted in a previous Letter to the Editor [12]), while others underdosed, possibly due to the absence of nociception monitoring. Nociception monitoring addresses only part of the perioperative stress response. Given the interindividual variability in drug needs, physiological stress monitoring remains promising but unvalidated in OFA and warrants further refinement. [13].

The implications extend well beyond pain control and hemodynamic stability. Preserving immune function is critical for preventing postoperative infection and supporting tissue repair. Avoiding immune suppression during anesthesia may also mitigate long-term complications increasingly linked to inflammatory pathways, including postoperative cognitive dysfunction (POCD) [14] and ventilator-induced lung injury (VILI) [15].

For these reasons, it seems premature to conclude, as Tripodi et al. do, that OFA has “no advantage.” On the contrary, the absence of inferiority in analgesia, combined with a more favorable side-effect profile, stronger preservation of immune function, and retrospective evidence of safety, strongly support further development and wider clinical implementation of opioid-free strategies. We agree that large, well-designed multicenter RCTs are needed to evaluate the role of OFA within multimodal anesthesia. Such trials should be preceded by structured education and training in OFA techniques [16], and supported by improved monitoring and protocol implementation, enabling precise, individualized titration of non-opioid agents. If intraoperative opioids offer no demonstrable benefit, the unavoidable question might also be: why continue to use them today?

If the current evidence for OFA still suffers from protocol-related variability in drug dosing, we expect that patient-centered, multicenter, and pragmatic implementation studies will clarify its true perioperative impact.

## Data Availability

No datasets were generated or analysed during the current study.

## References

[1] Tripodi G, Bugada D, Marotta C et al (2025) Effectiveness and safety of opioid-free anesthesia compared to opioid-based anesthesia: a systematic review and network meta-analysis. J Anesth Analg Crit Care 5:53 40804649 10.1186/s44158-025-00272-9 PMC12351777

[2] Bugada D, Lorini LF, Lavand’homme P (2021) Opioid free anesthesia: evidence for short and long-term outcome. Minerva Anestesiol 87:230–237 32755088 10.23736/S0375-9393.20.14515-2

[3] Brown EN, Pavone KJ, Naranjo M (2018) Multimodal general anesthesia: theory and practice. Anesth Analg 127:1246–1258 30252709 10.1213/ANE.0000000000003668 PMC6203428

[4] Mulier JP, Dillemans B (2019) Anaesthetic factors affecting outcome after bariatric surgery: a retrospective leveled regression analysis. Obes Surg 29:1841–1850 30879241 10.1007/s11695-019-03763-1

[5] Desborough JP (2000) The stress response to trauma and surgery. Br J Anaesth 85:109–117 10927999 10.1093/bja/85.1.109

[6] Cusack B, Buggy DJ (2020) Anaesthesia, analgesia, and the surgical stress response. BJA Educ 20:321–328 33456967 10.1016/j.bjae.2020.04.006 PMC7807970

[7] Liu S, Wu J, Wang Y et al (2024) Effect of regional vs general anesthesia on 30-day mortality in hip fracture surgery: a systematic review and meta-analysis. J Clin Anesth 93:111123

[8] Mufarrih SH, Sial AR, Khawaja S et al (2023) General vs regional anesthesia and postoperative outcomes: systematic review and meta-analysis. Am J Surg 226:1151–1161

[9] Xu S, Hu S, Ju X et al (2021) Effects of intravenous lidocaine, dexmedetomidine, and their combination on IL-1, IL-6 and TNF-α in patients undergoing laparoscopic hysterectomy: a prospective, randomized controlled trial. BMC Anesthesiol 21:36 33407156 10.1186/s12871-020-01219-z PMC7786488

[10] Kehlet H (1985) Stress-free anesthesia and surgery. Acta Anaesthesiol Scand Suppl 81:7–133909712

[11] Beloeil H, Garot M, Lebuffe G et al (2021) Opioid-free anesthesia with dexmedetomidine, lidocaine, and ketamine versus remifentanil-based anesthesia in major or intermediate noncardiac surgery: the POFA randomized clinical trial. Anesthesiology 134:541–551 33630043 10.1097/ALN.0000000000003725

[12] Forget P, Mulier J, Lavand’homme P, De Baerdemaeker L, Pelosi P, de Boer HD (2021) Opioid-free anesthesia: comment. Anesthesiology 135:751–753 34388823 10.1097/ALN.0000000000003908

[13] Snoek MAJ, van den Berg VJ, Dahan A et al (2025) Comparison of different monitors for measurement of nociception during general anaesthesia: a network meta-analysis of randomised controlled trials. Br J Anaesth 134:180–191 39609176 10.1016/j.bja.2024.09.020

[14] Subramaniyan S, Terrando N (2019) Neuroinflammation and perioperative neurocognitive disorders. Anesth Analg 128:781–788 30883423 10.1213/ANE.0000000000004053 PMC6437083

[15] Slutsky AS, Ranieri VM (2013) Ventilator-induced lung injury. N Engl J Med 369:2126–2136 24283226 10.1056/NEJMra1208707

[16] Blum KA, Liew LY, Dutia AR et al (2024) Opioid-free anesthesia: a practical guide for teaching and implementation. Minerva Anestesiol 90:300–310 38482635 10.23736/S0375-9393.23.17824-2

